# Supplementary material for: Mutation Hotspots Found in Bladder Cancer Aid Prediction of Carcinogenic Risk in Normal Urothelium
Source: Int J Mol Sci. 2023 Apr 25;24(9):7852. doi: 10.3390/ijms24097852 (PMC10177765; doi:10.3390/ijms24097852)
Supplement: Supplementary file 1 [file ijms-24-07852-s001.zip › ijms-2301128-supplementary.pdf]

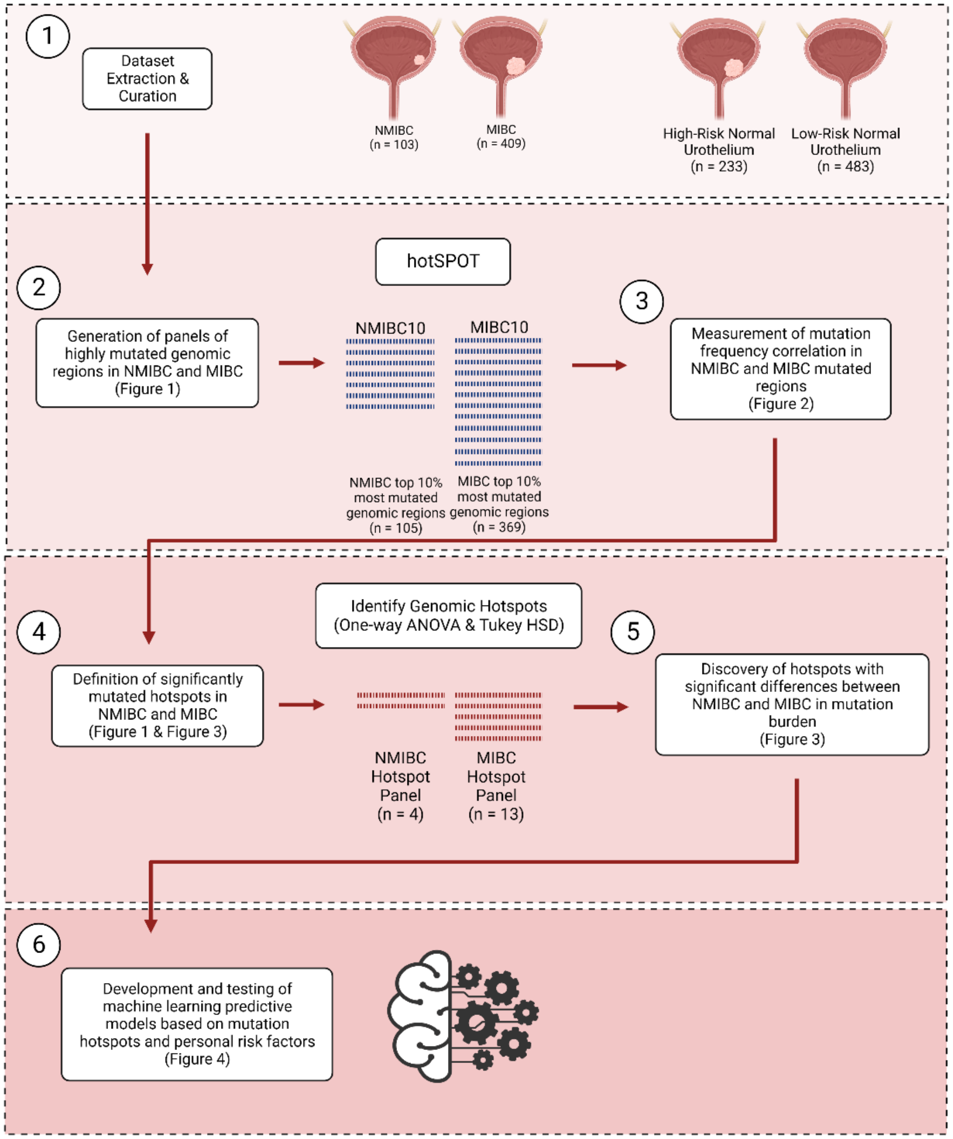

Supplementary Figure S1. Overview of study methodology.

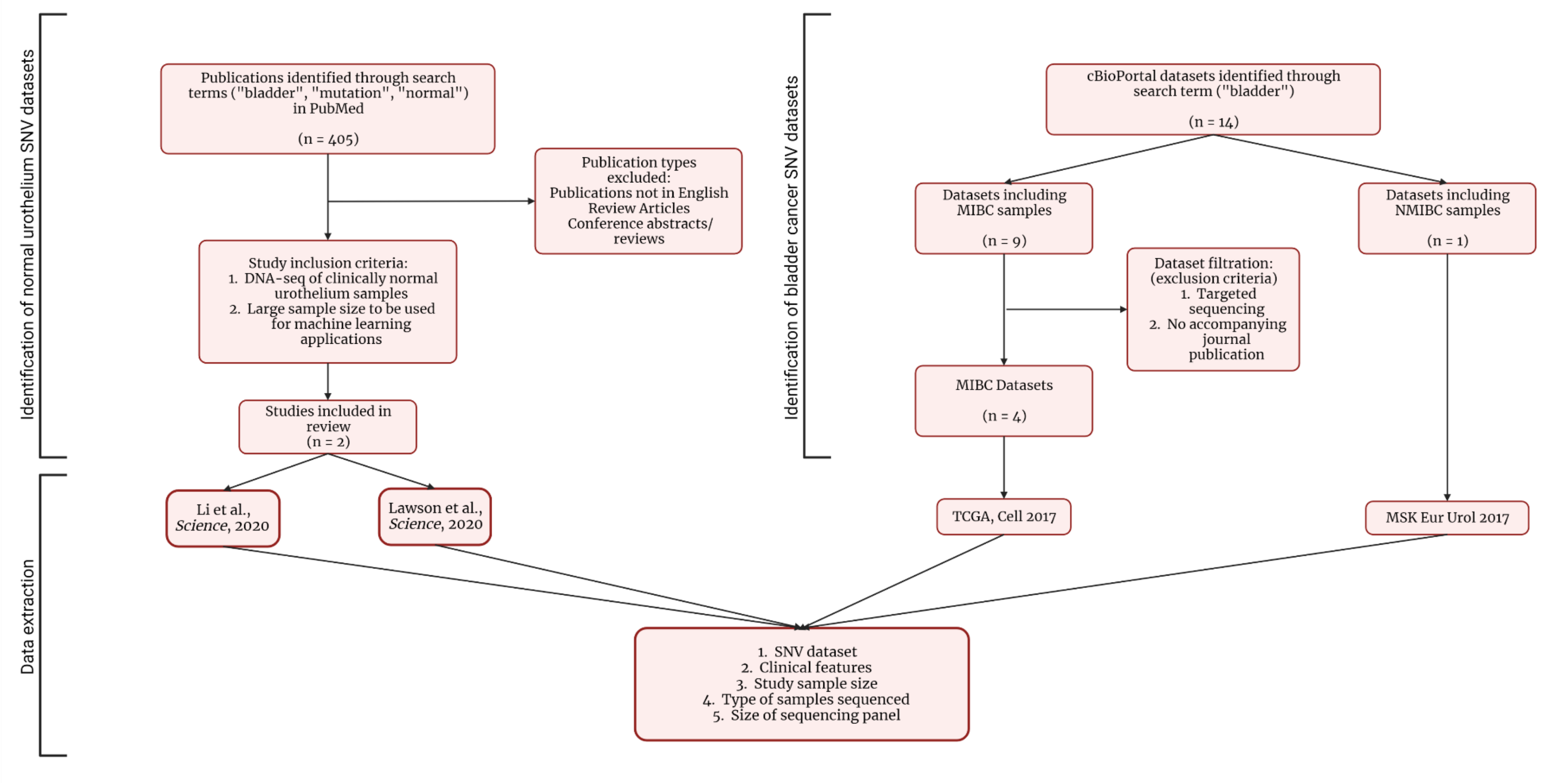

Supplementary Figure S2. Bladder cancer and normal urothelium dataset selection.

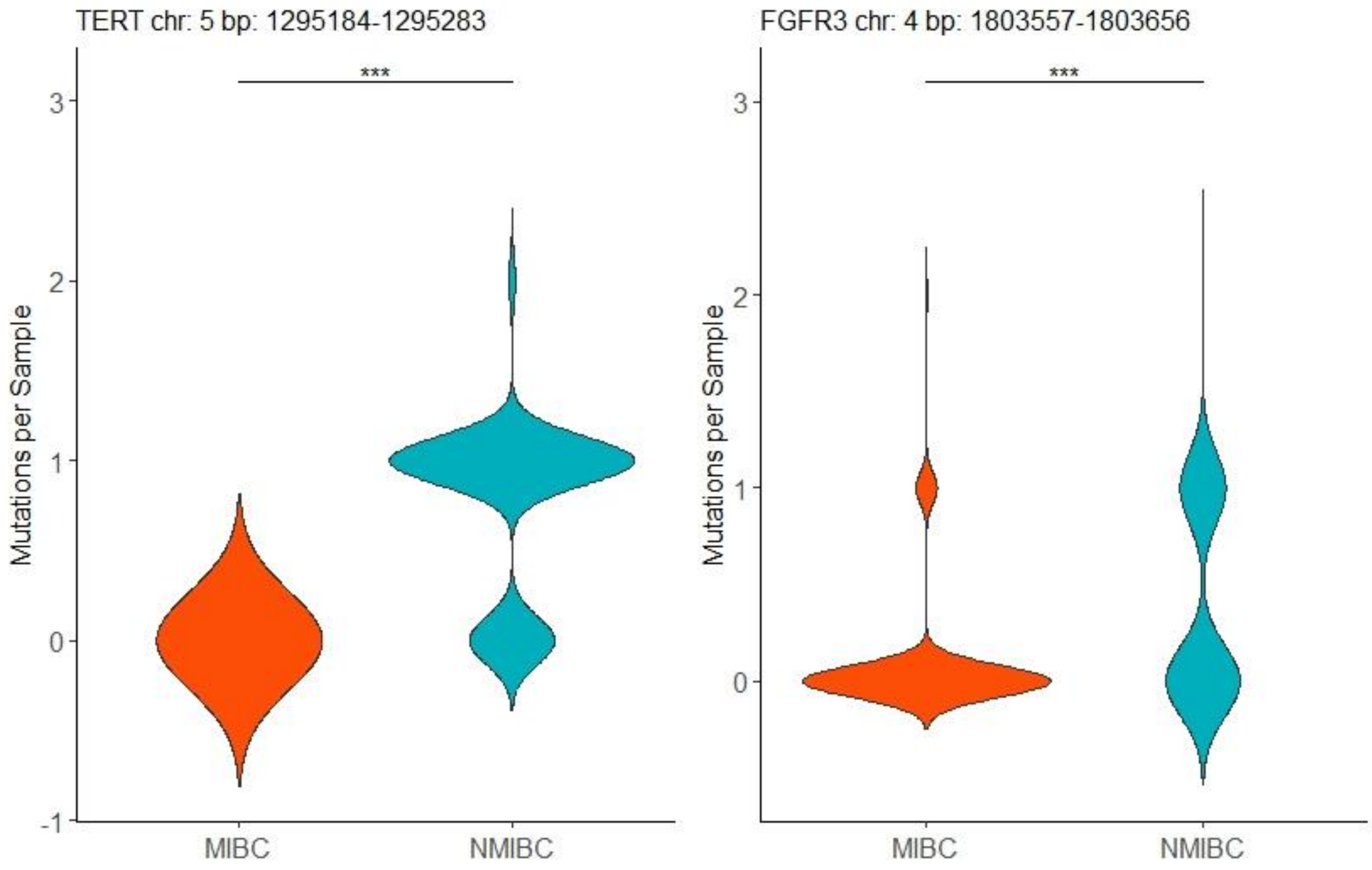

Supplementary Figure S3. Genomic segments with significantly higher mutation burden in NMIBC samples. Significance based on Wilcoxon signed-rank test. (***, $p < 0.001$).

Sample Type	Number of Samples	Sequencing Panel	Dataset Location
Muscle Invasive Bladder Cancer	412	WES	cBioPortal (TCGA, Cell 2017) PMID: 28988769
Non-Muscle Invasive Bladder Cancer	103	IMPACT targeted sequencing	cBioPortal (MSK Eur Urol 2017) PMID:28583311
Muscle Invasive Bladder Cancer	126	WES	PMID: 33004515
Low-Risk Normal Urothelium	483	WES	PMID: 33004514
High-Risk Normal Urothelium	72	WES	PMID: 33004514
High-Risk Normal Urothelium	161	WES	PMID: 33004515

Supplementary Table S1: Summary of publicly available datasets.

lowerbound	upperbound	chromosome	gene
1803519	1803618	4	FGFR3
178936038	178936137	3	PIK3CA
7577047	7577146	17	TP53
37868156	37868255	17	ERBB2

Supplementary Table S2: Genomic coordinates of NMIBC hotspot panel

lowerbound	upperbound	chromosome	gene
7577045	7577144	17	TP53
178936040	178936139	3	PIK3CA
7577494	7577593	17	TP53
1803557	1803656	4	FGFR3
7578380	7578479	17	TP53
7578183	7578282	17	TP53
37868159	37868258	17	ERBB2
36651972	36652071	6	CDKN1A
1806049	1806148	4	FGFR3
21971025	21971124	9	CDKN2A
178098876	178098975	2	NFE2L2
7578435	7578534	17	TP53
44922703	44922802	X	KDM6A

Supplementary Table S3: Genomic coordinates of MIBC hotspot panel
